# Boron Nitride as a Novel Support for Highly Stable Palladium Nanocatalysts by Atomic Layer Deposition

**DOI:** 10.3390/nano8100849

**Published:** 2018-10-18

**Authors:** Matthieu Weber, Cassandre Lamboux, Bruno Navarra, Philippe Miele, Sandrine Zanna, Maxime E. Dufond, Lionel Santinacci, Mikhael Bechelany

**Affiliations:** 1 Institut Européen des Membranes, IEM, UMR-5635, Univ Montpellier, CNRS, ENSCM, 34095 Montpellier, France; matthieu.weber@umontpellier.fr (M.W.); cassandrelamboux@gmail.com (C.L.); bruno.navarra@umontpellier.fr (B.N.); Philippe.Miele@umontpellier.fr (P.M.); 2 Institut Universitaire de France, 1 rue Descartes, 75231 Paris, France; 3 PSL Research University, Chimie ParisTech—CNRS, Institut de Recherche de Chimie Paris, 75005 Paris, France; sandrine.zanna@chimieparistech.psl.eu; 4 Aix Marseille Univ, CNRS, CINAM, 13009 Marseille, France; dufond@cinam.univ-mrs.fr (M.E.D.); lionel.santinacci@univ-amu.fr (L.S.)

**Keywords:** palladium, nanocatalysts, boron nitride, atomic layer deposition

## Abstract

The ability to prepare controllable nanocatalysts is of great interest for many chemical industries. Atomic layer deposition (ALD) is a vapor phase technique enabling the synthesis of conformal thin films and nanoparticles (NPs) on high surface area supports and has become an attractive new route to tailor supported metallic NPs. Virtually all the studies reported, focused on Pd NPs deposited on carbon and oxide surfaces. It is, however, important to focus on emerging catalyst supports such as boron nitride materials, which apart from possessing high thermal and chemical stability, also hold great promises for nanocatalysis applications. Herein, the synthesis of Pd NPs on boron nitride (BN) film substrates is demonstrated entirely by ALD for the first time. X-ray photoelectron spectroscopy indicated that stoichiometric BN formed as the main phase, with a small amount of BN_x_O_y_, and that the Pd particles synthesized were metallic. Using extensive transmission electron microscopy analysis, we study the evolution of the highly dispersed NPs as a function of the number of ALD cycles, and the thermal stability of the ALD-prepared Pd/BN catalysts up to 750 °C. The growth and coalescence mechanisms observed are discussed and compared with Pd NPs grown on other surfaces. The results show that the nanostructures of the BN/Pd NPs were relatively stable up to 500 °C. Consequent merging has been observed when annealing the samples at 750 °C, as the NPs’ average diameter increased from 8.3 ± 1.2 nm to 31 ± 4 nm. The results presented open up exciting new opportunities in the field of catalysis.

## 1. Introduction

Heterogeneous catalysis is at the heart of almost every established chemical process and is also crucial for the development of novel technologies in the fields of sustainable production and conversion of energy. Hence, different approaches have been developed to improve catalyst properties, aiming towards the promotion of catalytic reactions. Palladium nanoparticles (NPs) are known as very efficient catalysts, and the preparation of controllable and highly dispersed supported palladium NPs is therefore of huge interest for heterogeneous catalysis [1,2]. As the high surface-to-volume ratio of the particles is crucial in catalysis, palladium NPs with a narrow size distribution at the nanoscale are able to provide a high density of active sites available for catalysis. Therefore, enhancing atom efficiency and limiting the cost of this precious-metal catalyst [3,4,5]. In addition, since the catalytic activity of the metallic NPs depends on their size [6,7,8,9], the ability to synthesize stable and precisely engineered NPs in order to optimize their catalytic activity is highly desired. For example, in the case of alcohol oxidation, the intrinsic turnover frequency per surface Pd atom depends significantly on the nature of the substrates [10,11] as well as on the diameter of Pd NPs and showed a maximum at a medium size of 4 nm [6].

An important factor affecting the metal catalysts is their relative lack of thermal stability, resulting in nanomaterials that have a lower specific surface area due to sintering. In industrial catalysis, the sintering of NPs and the degradation of the supports are often associated with the loss of the catalyst activity, which must be avoided if possible [12]. Therefore, the metallic “active” phase needs to be stable and prepared on a thermally strong support phase in order to be used in “real life” under high operating temperature conditions. It is known that catalytic activity highly depends on the size and geometry of the NPs but also on the particle interactions with its supports [13]. The structural changes and the loss of active surface area due to the particles’ coalescence during operation lead to undesirable deactivation for supported catalysts, and new catalytic materials with enhanced stability must, therefore, be developed.

Typically, the support materials used for these metallic NPs are high melting point oxides and carbon-based materials [2,14,15]. However, as the design of catalytic nanomaterials with enhanced thermal stability is desired, it is important to focus on emerging catalyst supports such as boron nitride-based materials, which besides possessing extremely high thermal, mechanical, and chemical resistance [16,17,18,19], have also been recently investigated as hydrogen storage platforms and in nanocatalysis [20,21]. When compared to other support materials such as oxides and carbon, the potential of boron nitride (BN) as an innovative support for selective reactions has been demonstrated, especially for processes requiring high temperatures [22,23,24,25,26], and it has been shown that Pd NPs supported on BN open prospects for many catalytic reactions, such as the hydrogenation of lactose, for example [23,27,28,29].

Atomic layer deposition (ALD) is a vapor-phase deposition technique enabling the synthesis of ultrathin films of inorganic materials, such as oxides [30,31], nitrides [32,33,34,35], and metals [36,37], with a subnanometer thickness control [38,39]. ALD is based on the sequential use of self-limiting chemical reactions taking place in a cycle-wise manner, and a typical ALD cycle consists of alternate pulses of a precursor and co-reactant gases in the reactor, separated by purge or pumping steps. Obviously, each precursor or co-reactant in an ALD cycle has a profound impact on the process chemistry [38,40]. The benefits of the ALD method, especially the atomic-level thickness control, the excellent uniformity and conformality, enabled this route to emerge as an important technology for the deposition of thin films for a wide range of applications, from microelectronics [41] to biosensing [42] and from photovoltaics [43] to membranes [44]. For more information on this ALD technology, the reader is referred to excellent reviews already written [38,39,45,46,47].

The preparation of BN films by ALD is challenging, but several successful processes have been reported recently that enabled the deposition of BN with high uniformity and conformality [32,34,48,49,50,51,52,53,54]. Due to the difference of surface energies between the metal and the substrate surface [55], ALD of noble metals typically starts with the formation of isolated particles at the substrate surface, which grow and coalesce together with the increase of the number of cycles [56,57,58]. This peculiar nucleation-stage behavior can be used to prepare highly dispersed metallic NPs on high surface area supports and makes ALD a promising new route for the synthesis of nanocatalysts [56,57,59,60,61,62,63]. ALD of palladium NPs has been the subject of many studies, and the NPs have been deposited on different oxides, such as Al_2_O_3_ [37,64], TiO2 [65,66], ZrO2 [57], SiO2 [67], SnO2 [68], NiO [69] as well as on high aspect ratio carbon supports [70,71]. Furthermore, the catalytic activities of Pd NPs prepared by ALD have been assessed for different chemical reactions, such as ethanol [66]. Methanol [64], isopropanol [71], glucose [72] and glycerol oxidation [73].

The combination of Palladium NPs as active catalysts and BN as an innovative support material represent a very promising nanomaterial for heterogeneous catalysis. However, virtually all the Pd NPs synthesized by ALD have been prepared and studied on oxide and carbon supports. To the best of our knowledge, there is no work reporting on the preparation of palladium NPs by ALD on BN surfaces, despite its great potential. For the optimal use of these NPs, their structural changes and loss of active surface area due to particle coalescence need to be limited as much as possible. Recent studies have been dedicated to obtain a better understanding of the growth of metallic NPs by ALD, and to describe the pathways that lead from adsorbed single atoms to supported palladium NPs [56,74,75]. Although the growth of Pd NPs by ALD on oxides and their potential as active nanocatalysts for various chemical reactions have been reported, there is little understanding about Pd NPs on BN supports, nor about the stability of the NPs at high temperatures.

Herein, we synthesize this new class of Pd NPs/BN nanomaterials entirely by ALD, and we present a detailed study of the Pd NPs growth on BN surfaces. First, ALD of Pd and BN processes have been developed, and the preparation of BN films and Pd NPs is achieved. Using transmission electron spectroscopy (TEM) data, the evolution of the palladium NPs diameter as a function of the number of ALD cycles is presented, and the pathways leading from adsorbed single atoms to supported metallic NPs are discussed. Our data is compared to the data obtained from the literature in order to shed light on and to understand the growth of Pd NPs on BN surfaces. Furthermore, we study the chemical composition by X-ray photoelectron spectroscopy (XPS) as well as the thermal stability of the NPs, up to 750 °C. The coalescence mechanisms observed are discussed and compared with Pd NPs grown by ALD on other surfaces.

## 2. Materials and Methods

### 2.1. Atomic Layer Deposition of Boron Nitride

All depositions have been carried out in a low-pressure hot-wall (home-built) ALD reactor. More details about this ALD reactor can be found in reference [34]. Boron tribromide (BBr_3_) precursor was purchased from Sigma Aldrich (Saint-Louis, MO, USA) and used as received. The co-reactant was ammonia gas (Air Liquide, Paris, France). The precursor and co-reactant lines were directly connected to the reactor through gate valves and heated at 110 °C to avoid condensation. The deposition chamber was set at a temperature of 750 °C. If not stated otherwise, the typical ALD cycle consisted of 0.1 s pulse of BBr_3_, 5 s exposure, and 15 s purge, followed by a 3 s pulse of NH_3_, 5 s exposure, and 15 s purge with Argon (Air Liquide, Paris, France). The silicon nitride TEM window grid substrates were 20 nm thick and purchased from Electron Microscopy Sciences (EMA, Hatfield, PA, USA). The p-type (100) silicon wafer substrates were purchased from MEMC Korea Company (Cheonan, South Korea). To remove the organic contaminants, the substrates were pre-cleaned in acetone, ethanol, and de-ionized water for 5 min in an ultrasonic bath before the depositions.

### 2.2. Atomic Layer Deposition of Palladium

All depositions have been done in a low-pressure hot-wall (home-built) ALD reactor, described elsewhere [73]. The precursor Pd(hfac)_2_ and co-reactant formalin were purchased from Sigma Aldrich (Saint-Louis, MO, USA). These precursor lines were heated at 70 °C and 100 °C for Pd(hfac)_2_ and formalin, respectively, to avoid condensation. The deposition chamber was set at a temperature of 220 °C. If not stated otherwise, the typical ALD cycle consisted of a 5 s pulse of Pd(hfac)_2_, 15 s exposure, and 20 s purge, followed by a 1 s pulse of formalin, 15 s exposure, and 60 s purge with Argon.

### 2.3. Thermal Treatments

The thermal treatments have been carried out directly within the ALD reactor used for BN ALD, for 3 h under Ar gas, using a fast ramp rate of 45 °C/min.

### 2.4. Transmission Electron Microscopy Imaging and Analysis of the Palladium NPs

A JEOL 3010 high-resolution transmission electron microscope at 300 kV (JEOL, Tokyo, Japan) has been used for the HR-TEM studies. The number of NPs, size distribution, and surface coverage were determined over areas of 400 × 400 nm^2^ using the ImageJ software (NIH, MD, USA). The average diameter and its standard deviation were determined by averaging ten analyses of areas of 400 × 400 nm^2^.

### 2.5. Chemical Analysis

XPS analysis was performed using a VG ESCALAB 250 spectrometer (ThermoFisher Scientific, Waltham, MA, USA) calibrated against the reference binding energies (BE) of clean Cu (932.6 eV), Ag (368.2 eV), and Au (84 eV) samples. Survey spectra and high-resolution spectra of the C 1s, B 1s, N 1s, O 1s, and Pd 3d core-level regions were collected at a take-off angle of 90° and a pass energy of 100 and 20 eV, respectively, using an Al K_α_ monochromated X-ray source (hν = 1486.6 eV). Data processing was performed using the CasaXPS analysis software using a Shirley-type background, component peaks defined by BE, Full Width at Half Maximum (FWHM) and Gaussian/Lorentzian envelopes. BEs of the component peaks were corrected with reference to the C 1s peak for –CH_2_—CH_2_– bonds set at 285.0 eV.

## 3. Results and Discussion

First, an ALD process for the preparation of BN thin films has been employed to coat the TEM window grid substrates. The process consisted of sequential exposures of boron tribromide precursor (BBr_3_) and NH_3_ gas, separated by purge steps of Argon at a temperature of 750 °C. The BN films have been prepared (and measured by spectroscopic ellipsometry) on silicon with native oxide substrates in parallel to ensure the success of the ALD process. This process leads to the saturated growth of high quality BN thin films with a steady-state ALD growth of ~0.8 Å/cycle and no visible nucleation delay, as measured by spectroscopic ellipsometry [34,58,76]. Our process is based on the recipe developed by Marlid et al. [32] which studied the growth at different temperatures and showed that this ALD process based on BBr_3_ and NH_3_ enables the deposition of good quality BN films from 400 to (at least) 750 °C, depicting the large temperature “ALD windows”. As the microstructure observed at 750 °C was better than that which was observed at lower temperature, our process was applied at this temperature. The saturation curve of the BN film growth as a function of the BBr_3_ exposure time, depicting the saturated growth of the film, is given in the Supplementary Materials, Figure S1. Owing to the linearity of the process, the thickness of the prepared layers can be easily tuned. The detailed parameters of this process are given in the Experimental section, and more information can also be found elsewhere [34,53,77]. Several analytical methods have been used to characterize the BN films, and the growth-per-cycle, C and O contents, mass density, and roughness values are given in Table S1 (Supplementary Materials). In this study, in order to investigate the nanoparticles’ growth on BN surfaces, we applied 200 cycles of this ALD process to coat Si_3_N_4_ TEM window substrates with a BN film of approximately 15 nm. Next, we used ALD of Palladium to deposit the Pd NPs on the BN surface. ALD of Palladium was based on Pd(hfac)_2_ and formalin precursors separated by Ar purged at 220 °C. The typical ALD cycle consisted of 5 s pulse of Pd(hfac)_2_, 15 s exposure, and 20 s purge, followed by a 1 s pulse of formalin, 15 s exposure, and 60 s purge with Argon. The detailed process parameters are indicated in the Experimental section. Note that we already reported the use of this process for the preparation of Pd NPs by ALD on high aspect ratio carbon fibers, as well as their enhanced catalytic activity for glycerol oxidation [73].

**Figure 1:**
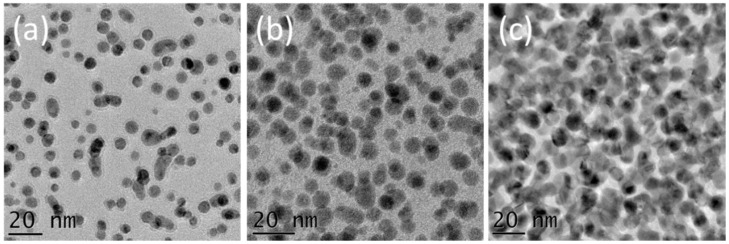
Transmission electron microscopy (TEM) images of boron nitride (BN) surfaces after (**a**) 100, (**b**) 200, and (**c**) 300 cycles of the Pd atomic layer deposition (ALD) process. The substrates used were Si_3_N_4_ TEM windows covered with 15 nm of BN.

In this work, in order to gain understanding on the formation and the growth of Pd NPs on BN surfaces, the BN coated TEM windows were used as substrates and 100, 200, and 300 cycles of Pd ALD cycles have been applied. TEM measurements have been performed in order to obtain information on the nanomaterials prepared. The data shown in Figure 1a–c presents the TEM images of the BN/Pd NPs as a function of the number of Pd ALD cycles.

**Figure 2:**
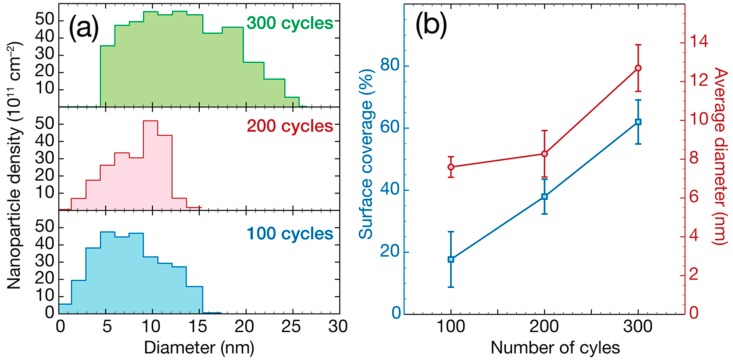
(**a**) Nanoparticle (NP) size distribution and (**b**) average diameter and surface density evolution of the Pd NPs on BN surfaces after 100, 200 and 300 ALD cycles (estimated). The data analysis is based on the TEM images presented in Figure 1.

This figure illustrates that the particles’ nucleation involves successive steps. Most of the Pd NPs are formed during the first 100 cycles. TEM data analysis, shown in Figure 2, revealed a surface density of ~10^13^ NPs/cm^2^ and a surface coverage of 18% ± 9%. After 200 cycles, more NPs are formed all over the substrate and start to merge, reaching a surface coverage of 38% ± 6%. The surface density increased to 4 × 10^13^ NPs/cm^2^. After 300 cycles, most of the NPs are merged, which can be described as particle coalescence. The surface coverage after 300 cycles is around 62% ± 14%. The difference in gray color between the NPs in the TEM images is related to the diffraction contrast, indicating that the deposited NPs are crystalline. In order to confirm this crystallinity, diffraction studies have been performed on a BN/Pd NPs sample prepared using 300 cycles of the Pd ALD process, sample shown in Figure 1c. Various crystal planes have been observed, as revealed by the selected area electron diffraction study carried out, see Figure S2 (Supplementary Materials).

Figure 2a presents the NP size distribution, and Figure 2b shows the average diameter evolution for the Pd ALD process on BN surfaces after 100, 200, and 300 ALD cycles.

After 100 cycles, the NPs present an average diameter of 7.7 ± 1.3 nm. At 200 cycles, more Pd material is deposited and the average diameter value increased to 8.3 ± 1.2 nm. Finally, after 300 cycles, since the NPs are mostly merged, it is challenging to evaluate an average NP diameter, and we estimated it to be around 12.6 ± 2.2 nm. From this data, it can be deduced that the diameter of the Pd NPs increases with a growth rate of ~0.02 nm/cycle between 100 and 300 cycles, which corresponds to a radial growth rate of ~0.01 nm/cycle. This radial growth is two times lower than the rate observed for film growth using the same process (~0.02 nm/cycle) [78]. However, the chemical reactions during ALD nucleation are different from the reactions taking place during film growth. The slow growth rate of the NPs observed could be explained by surface ligand pollution inhibiting the growth of the particles. This ligand pollution is known to be responsible for the long nucleation periods typically observed for ALD using precursors based on β-diketonates [37,79,80]. In fact, studies carried out previously have shown that Pd(hfac)_2_ adsorption (on alumina surfaces) results in both Pd(hfac)* and Al(hfac)* surface species. The Al(hfac)* species appeared to act as site blockers for precursor adsorption. This surface poisoning by hfac ligands has been found to be partly responsible for the extended nucleation delay observed for Pd ALD on alumina [79,80]. However, similar poisoning is also expected for other surfaces such as nitrides, since the nucleation is also supposed to start at hydroxyl groups.

**Figure 3:**
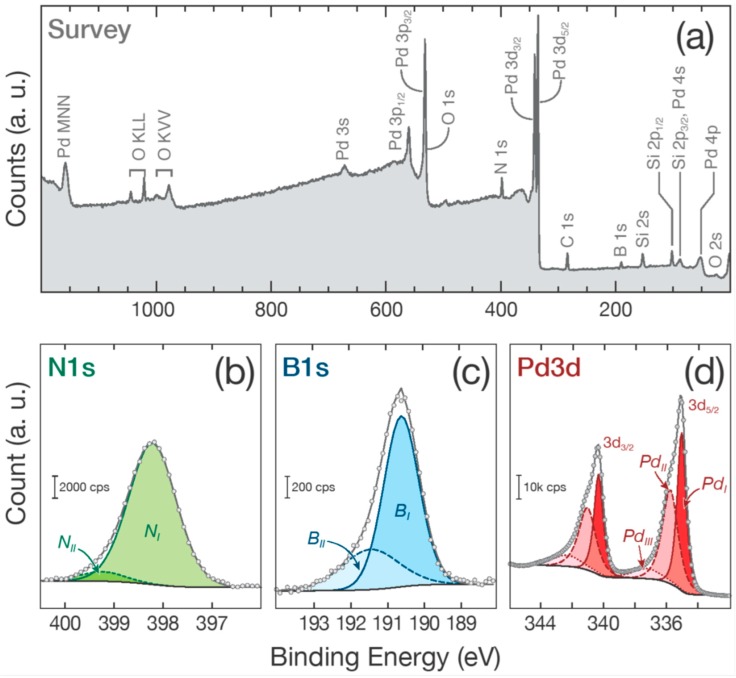
(**a**) XPS survey of the BN/Pd NPs sample. (**b**–**d**) present the N 1s, B 1s, and Pd 3d deconvoluted peaks, respectively.

The surface composition of the BN/Pd nanomaterials deposited on Si wafers has been investigated by X-ray photoelectron spectroscopy. The XPS survey is presented in Figure 3a. After the ALD process, B, C, N, O, Si, and Pd are detected. It indicates that the B, N, and Pd have effectively been deposited on the Si surface. The C and O could correspond to surface contamination and oxidation. As expected, no other elements are observed. The quantification indicates a B/N ratio of 1.08 that is in line with the value of 1.13 when the BN is obtained from trichloroborazine [81]. Though it is not far from the unity it indicates that non-stoichiometric BN has been grown or that those elements are part of other compounds. Thus, the regions of interest have been investigated with a higher resolution in order to identify precisely the chemical nature of the film.

Figure 3b–d presents the N 1s (b), B 1s (c), and Pd 3d (d) peaks. The peak deconvolutions performed give valuable information on the chemical nature of the sample after the ALD of BN and Pd. The N 1s peak is deconvoluted using two contributions *N_I_* and *N_II_*. At low binding energy (BE = 398.2 eV), the *N_I_* peak is expected because it corresponds to nitrogen in BN [82]. *N_II_* located at higher BE (399.2 eV) can be attributed to the formation of BN_x_O_y_. It could indicate that a minor part of the BN reacts with oxygen to form a small amount of boron oxynitride. Similarly, the B 1s peak is fitted using two contributions: *B_I_* at 190.6 eV and *B_II_* at 191.4 eV. The *B_I_* contribution is ascribed to boron in BN [82] while *B_II_* could correspond to BN_x_O_y_. This has been reported before [81] and is confirmed by the deconvolution of the O 1s peak (not shown here) that is composed of four contributions: 530 eV for PdO/PdO_2_, 531.8 eV for the Pd 3p peak, 532.8 eV for adsorbed oxygen and H_2_O, and 535.15 eV for BN_x_O_y_. As the Pd ALD process is expected to start with the reaction between the Pd(hfac)_2_ precursor molecules and hydroxyl groups, the additional OH groups created during the surface oxidation could actually aid the palladium nucleation. The deconvolution of the Pd 3d peak. see Figure 3d, reveals that beyond the component at lower BE *Pd_I_* (Pd 3d_5/2_: 335.1 eV) due to metallic Pd [82], two additional peaks (*Pd_II_* and *Pd_III_*) are present at higher BEs (335.8 and 337.3 eV) [83]. These two peaks are attributed to palladium in the 2+ and 4+ oxidation states due to PdO and PdO_2_ and/or respective hydroxides.

The data obtained can also be compared to results reported in the literature for other substrates. Assaud et al. used ALD of palladium on TiO_2_ surface for electrocatalytic applications and reported NPs of 6–8 nm after 400 cycles using a similar process based on Pd(hfac)_2_ and formalin [66]. Goldstein and George studied palladium ALD nucleation on an alumina surface and obtained NPs with an average diameter of 9.6 ± 2.0 nm after 150 cycles using a similar process based on Pd(hfac)_2_, trimethyl alumina, and formalin [79]. Mackus et al. studied the nucleation of Pd ALD on Al_2_O_3_ surfaces and observed the long nucleation delay and slow growth rate of NPs as well. They found that the average diameter of the Pd NPs after 200 cycles was around 5 nm and increases with a rate of ~0.014 nm/cycle [56]. Only Barr et al. have not found a long nucleation delay on SnO_2_, most likely because the formalin reduces the stannic oxide to metallic tin which exhibits a better interaction with Pd than the oxide [68]. Although the ALD processes used for these results on alumina were slightly different from ours, the values are comparable to the average diameter after 200 cycles (8.4 ± 1.4 nm) and diameter growth of ~0.02 nm/cycle that we obtained in our work where ALD of Pd was carried out on a boron nitride surface.

The fact that the diameter of the NPs slowly increases with the number of cycles brings a certain control on the tuning of the NPs by choosing the appropriate number of ALD cycles. The control of NPs dimensions is very important for heterogeneous catalysis applications. Many different parameters such as pressure, substrate temperature, and co-reactant exposure are involved in their growth but the formation of metallic NPs by ALD is mainly based on the difference in the surface energies between the metal and the substrate surface, diffusion processes, and the ALD precursors’ chemistries [56,74,75,84].

**Figure 4:**
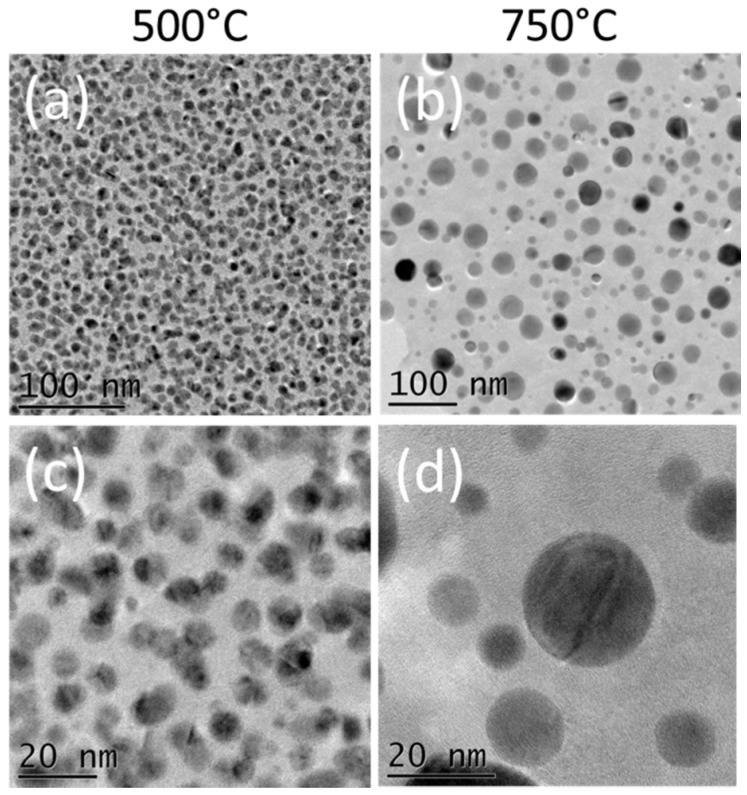
TEM images of Pd NPs on BN surfaces prepared by applying 200 cycles of the Pd ALD process, after heating treatments at (**a**,**c**) 500 °C and (**b**,**d**) 750 °C for 3 h. The substrates used were Si_3_N_4_ TEM window grids covered with 15 nm of BN.

As discussed in the introduction, highly dispersed NPs presenting a narrow size distribution are desired in order to obtain the largest density of active sites potentially available for catalysis. Using our Pd ALD process on a BN surface, the samples obtained using 200 cycles of Pd ALD appear to be optimized, presenting a high surface density of 4 × 10^13^ NPs/cm^2^. This surface density value is comparable to the typical values obtained for Pd or Pt NPs on oxide surfaces (10^12^–10^13^ NPs/cm^2^) [55,56,74,75]. The structural changes and the loss of active surface area due to the particle coalescence during industrial operation lead to undesirable deactivation for supported catalysts. Therefore, thermally stable catalytic materials are desired. Therefore, the stability of the synthesized BN/Pd NPs catalysts under high-temperature conditions has been studied. For this purpose, the BN/Pd NPs obtained using 200 cycles were submitted to different heat treatments. In order to carry out the thermal stability study, the samples prepared with 200 cycles were heated to 500 and 750 °C for 3 h (under constant Ar gas flow). Figure 4 presents TEM images of the Pd NPs/BN catalysts after heating treatments at 500 and 750 °C for 3 h.

For NPs on any surfaces, there are two limiting cases of the kinetics of dimensional changes: (i) Coalescence, in which NPs adhere poorly to the surface, permitting them to diffuse across it and to coalesce; (ii) Ostwald ripening, in which the NPs adhere strongly to the surface, making atomic transfer between NPs more favorable. The Ostwald ripening process is the main form of thermal annealing for metallic NPs that are well separated and supported on a surface, although coalescence can occur for a high density of clusters [55,85]. From Figure 4a,c, it can be seen that the NPs annealed at 500 °C present a very similar morphology to the as-deposited ones at 220 °C, see Figure 1b. A slight increase in the average diameter is however observed. TEM data analysis showed that this diameter increased from 8 nm to 11 nm. As expected, this merging of NPs resulted in a decrease in surface coverage, which was reduced from 38% ± 6% to 28% ± 3% after heat treatment at 500 °C when compared to the Pd NPs prepared at 220 °C. From the small shift in size distribution, a profile skewed more toward larger particles can be seen, suggesting that Oswald ripening took place as the main mechanism for the formation of larger NPs. However, these changes are expected to be relatively unharmful for potential catalysis applications, since the NPs diameter remains small and narrow, and the surface density remains high (value of 4 × 10^13^ NPs/cm^2^).

**Figure 5:**
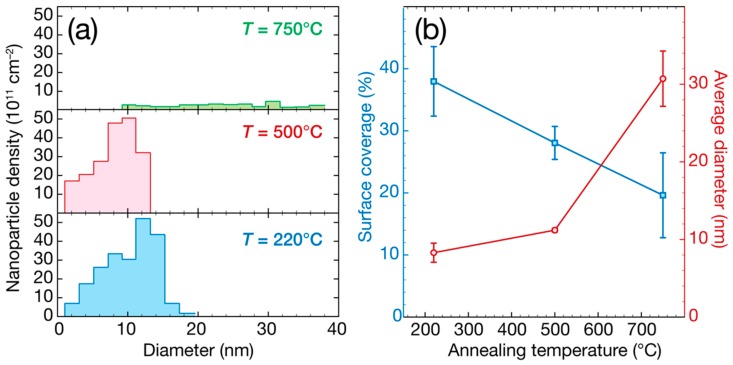
(**a**) Nanoparticle size distribution and (**b**) average diameter and surface coverage evolution of the Pd NPs on BN surfaces, as prepared at 220 °C and after thermal treatments at 500 and 750 °C for 3 h. The data analysis is based on the TEM images presented in Figure 4.

After annealing at 750 °C, the morphology of the NPs changes considerably. First, consequent merging between the NPs can be seen, and it can be observed as well that the NPs’ surface becomes slightly smoother, see Figure 4b,d. As revealed from the TEM data analysis presented in Figure 5, the average diameter increases to 31 ± 4 nm, whereas the surface coverage is reduced to 20% ± 7%. Furthermore, the density of NPs decreases by an order of magnitude to 10^12^ NPs/cm^2^. The enthalpy of activation of the coalescence between Pd NPs is directly dependent on the temperature [85], and 750 °C is in this case sufficient for this mechanism to take place at a “large” scale. Once the NPs adhere poorly to the surface and are able to diffuse across it, this coalescence mechanism is in fact favorable, as its driving force is the surface energy reduction (because the surface area of the new NP is less than that of the sum of the surface areas of the original smaller NPs). Due to the high mobility of Pd atoms on the high-temperature BN surface, surface-mediated Ostwald ripening sintering, in which material is transferred from one NP to another by diffusion across the substrate surface, is also very likely to take place, as a competitive process. The fact that the surface of the NPs seems spherical and smooth also indicates an easy diffusion of Pd atoms at the surface of the NPs, forming, thermodynamically speaking, a more stable NP shape.

From these results, it can be concluded that up to 500 °C, the morphology of the Pd NPs supported on BN remain relatively stable, opening opportunities for their use as catalysts at this temperature. However, consequent merging between the NPs has been observed for the annealed sample at 750 °C, due to the diffusion of atoms and NPs at this high temperature. These data allow for insights into the underlying mechanism of supported palladium NPs on BN supports to be gained, paving the way toward the rational design of this novel type of supported catalysts with controlled activity and stability.

## 4. Conclusions

In this work, we reported the synthesis of BN/Pd nanomaterials entirely by ALD. The thickness of the BN support and the size of the Pd NPs are easily controllable by varying the number of ALD cycles applied. After 200 cycles, the NPs were highly dispersed (10^13^ NPs/cm^2^) and presented an average diameter of 8.3 ± 1.2 nm. X-ray photoelectron spectroscopy analysis indicated that the Pd NPs were metallic and prepared on stoichiometric BN. The thermal stability study carried out revealed that the ALD-prepared Pd NPs supported on BN surface were relatively stable up to 500 °C and covered approximately 28% of the surface, but their morphology drastically changed when heated at higher temperatures. At 750 °C, the NPs were involved in a merging process, and their average diameter consequently increased up to 31 nm, whereas the surface coverage and dispersion were reduced to 20% and 10^12^ NPs/cm^2^, respectively. This study brings more understanding on the growth and thermal stability of this new type of BN/Pd NPs, which is valuable for their potential use as nanocatalysts at high temperature and opens up novel opportunities in the field of heterogeneous catalysis.

## Supplementary Materials

The following are available online at http://www.mdpi.com/2079-4991/8/10/849/s1. Table S1: Properties of BN films prepared by ALD using BBr_3_ as precursor and NH_3_ as co-reactant at 750 °C. Figure S1: Saturation curve of the BN film growth as a function of the BBr3 exposure time, depicting the saturated growth of the film. Figure S2: Selected area electron diffraction of a BN/Pd NPs sample prepared using 300 cycles of the Pd ALD process, corresponding to Figure 1c. The substrates used were Si_3_N_4_ TEM windows covered with 15 nm of BN. The table corresponds to the indexing of the diffraction rings.
