# Supplementary material for: Boron Nitride as a Novel Support for Highly Stable Palladium Nanocatalysts by Atomic Layer Deposition
Source: Nanomaterials (Basel). 2018 Oct 18;8(10):849. doi: 10.3390/nano8100849 (PMC6215320; doi:10.3390/nano8100849)
Supplement: Supplementary file 1 [file nanomaterials-08-00849-s001.pdf]

Supporting Information

Boron Nitride as a novel support for highly stable Palladium nanocatalysts by Atomic Layer Deposition

Matthieu Weber,[1] Cassandre Lamboux,[1] Bruno Navarra,[1] Philippe Miele,[1,2] Sandrine Zanna,[3] Maxime Dufond,[4] Lionel Santinacci,[4] Mikhael Bechelany[1,*]

1 Institut Européen des membranes, IEM, UMR-5635, Univ Montpellier, CNRS, ENSCM, Montpellier, France.
2 Institut Universitaire de France, 1 rue Descartes, 75231 Paris, France.
3 PSL Research University, Chimie ParisTech - CNRS, Institut de Recherche de Chimie Paris, 75005, Paris, France.
4 Aix Marseille Univ, CNRS, CINAM, Marseille, France.
* Correspondence: mikhael.bechelany@umontpellier.fr;

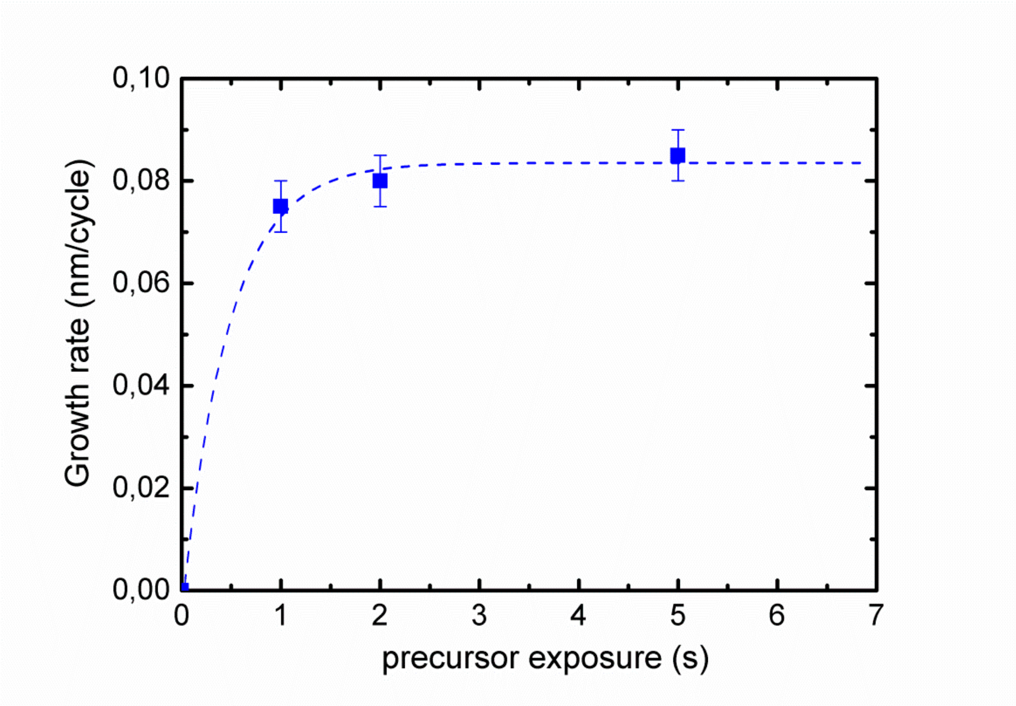

Figure S1: Saturation curve of the BN film growth as a function of the BBr_3 exposure time, depicting the saturated growth of the film.

Table S1: Properties of BN films prepared by ALD using BBr_3 as precursor and NH_3 as co-reactant at 750°C. The substrates were Si(100) wafers and the substrate temperature was 750°C. In situ spectroscopic ellipsometry (SE), X-Ray reflectometry (XRR), atomic force microscopy (AFM), X-Ray photoelectron spectroscopy (XPS) measurements were used for the analysis. The films were 25 nm thick for all analysis.

**Properties**	**Value**	**Analysis technique**
Growth-per-cycle (Å/cycle)	0.8±0.1	SE
Mass density (g/cm^3)	2.2±0.3	XRR
RMS roughness (nm)	3.5±2	AFM
C content (at.%)	4±3	XPS
O content (at.%)	7±3	XPS

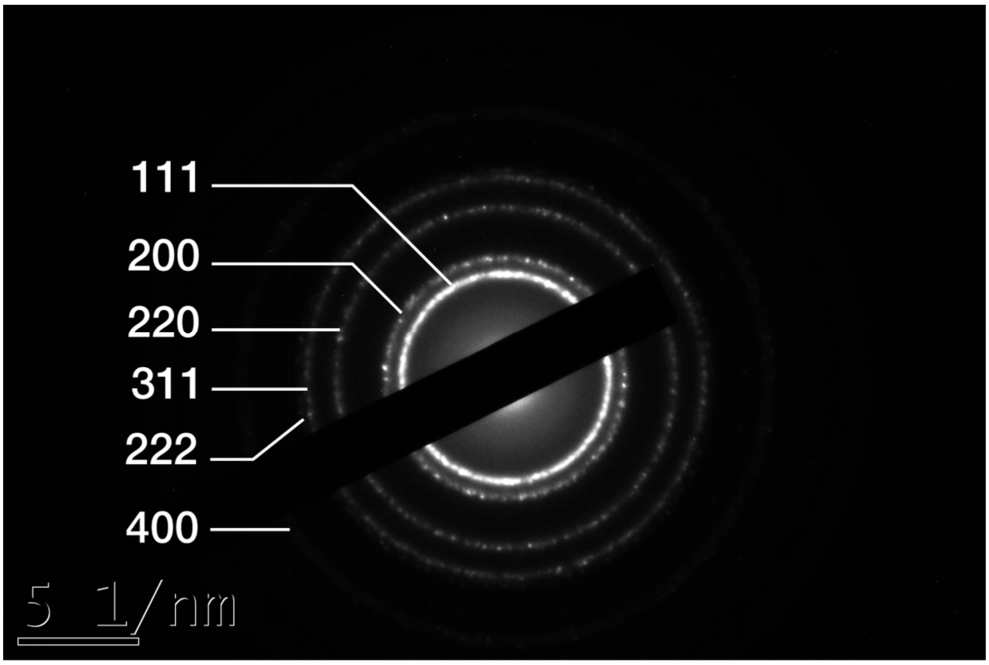

Crystal	*Diameter of the diffraction rings (nm^{-1})*	*d (Å)*	*Crystal planes*
Pd	*8.46*	*2.36*	*111*
	9.90	*2.02*	*200*
	13.93	*1.44*	*220*
	16.34	*1.22*	*311*
	17.21	*1.16*	*222*
	21.86	*0.91*	*400*

Figure S2: Selected area electron diffraction of a BN/Pd NPs sample prepared using 300 cycles of the Pd ALD process, corresponding to Figure 1c. The substrates used were Si_3N_4 TEM windows covered with 15 nm of BN. The table corresponds to the indexing of the diffraction rings.
